# Cathepsin L in COVID-19: From Pharmacological Evidences to Genetics

**DOI:** 10.3389/fcimb.2020.589505

**Published:** 2020-12-08

**Authors:** Caio P. Gomes, Danilo E. Fernandes, Fernanda Casimiro, Gustavo F. da Mata, Michelle T. Passos, Patricia Varela, Gianna Mastroianni-Kirsztajn, João Bosco Pesquero

**Affiliations:** ^1^ Center for Research and Molecular Diagnostic of Genetic Diseases, Department of Biophysics, Federal University of São Paulo, São Paulo, Brazil; ^2^ Division of Nephrology, Department of Medicine, Federal University of São Paulo, São Paulo, Brazil

**Keywords:** COVID-19, cathepsins, cathepsin L, SARS-CoV-2, pandemics

## Abstract

The coronavirus disease 2019 (COVID-19) pandemics is a challenge without precedent for the modern science. Acute Respiratory Discomfort Syndrome (ARDS) is the most common immunopathological event in SARS-CoV-2, SARS-CoV, and MERS-CoV infections. Fast lung deterioration results of cytokine storm determined by a robust immunological response leading to ARDS and multiple organ failure. Here, we show cysteine protease Cathepsin L (CatL) involvement with severe acute respiratory syndrome coronavirus 2 (SARS-CoV-2) and COVID-19 from different points of view. CatL is a lysosomal enzyme that participates in numerous physiological processes, including apoptosis, antigen processing, and extracellular matrix remodeling. CatL is implicated in pathological conditions like invasion and metastasis of tumors, inflammatory status, atherosclerosis, renal disease, diabetes, bone diseases, viral infection, and other diseases. CatL expression is up-regulated during chronic inflammation and is involved in degrading extracellular matrix, an important process for SARS-CoV-2 to enter host cells. In addition, CatL is probably involved in processing SARS-CoV-2 spike protein. As its inhibition is detrimental to SARS-CoV-2 infection and possibly exit from cells during late stages of infection, CatL could have been considered a valuable therapeutic target. Therefore, we describe here some drugs already in the market with potential CatL inhibiting capacity that could be used to treat COVID-19 patients. In addition, we discuss the possible role of host genetics in the etiology and spreading of the disease.

## Introduction

The coronavirus disease 2019 (COVID-19) pandemics is a challenge without precedent for the modern science. Worldwide scientists and health professionals are currently looking for feasible solutions to fight this disease, by testing medications and vaccines or looking for understanding its mechanisms. In the present paper we show the involvement of Cathepsin L (CatL) in severe acute respiratory syndrome coronavirus 2 (SARS-CoV-2) from different points of view.

## The COVID-19

In December 2019, a group of patients with pneumonia of unknown cause was observed in Wuhan, China (Zhu et al., 2020) On December 31, 2019, the Chinese Center for Disease Control and Prevention (China CDC) conducted an epidemiologic and etiologic investigation and China's authorities had reported to World Health Organization (WHO) about the disease (Wu and McGoogan, 2020; Zhu et al., 2020). Chinese scientists identified the pathogen as a novel coronavirus in January, 7, 2020, and a few weeks later, in January, 30, 2020, WHO declares a “public health emergency of international concern” (Wu and McGoogan, 2020). Coronavirus were first identified in 1968 by a group of virologists and named after their characteristic appearance in electron microscopy resembling a solar corona (Almeida et al., 1968). On February 11, 2020, WHO officially named the disease as coronavirus disease 2019 (COVID-19) and Coronavirus Study Group (CSG) of the International Committee proposed to name the new coronavirus as SARS-CoV-2 (Guo et al., 2020). The disease continues at an overwhelming rate, with more than 4.5 million cases and more than 300,000 deaths worldwide (Coronavirus Resource Center, 2020).

Until the present moment, the disease has no predilection for race and most patients (87%) were between 30 and 79 years old, in China (Wu and McGoogan, 2020). Recently, there are descriptions of cases in children, named Kawasaki-like disease, that need more studies to confirm the relation with SARS-COV-2 infection (Viner and Whittaker, 2020).

COVID-19 presents with a wide spectrum of clinical manifestations and it varies from asymptomatic and oligosymptomatic cases to critical illness, requiring intensive therapy and mechanical ventilation. COVID-19 can evolve in three patterns: mild disease, which corresponds to 80% to 85% of cases, with manifestation of fever, cough, sore throat, fatigue, malaise, headache, myalgia, without evidence of pneumonia. In general, these symptoms are self-limiting and there is no need for hospitalization or interventions. There are mild symptoms despite high viral load. The second pattern (10–15% of the cases) corresponds to moderate/severe disease or biphasic disease in which, in addition to mild symptoms, there is pulmonary involvement, with pneumonia and extension of the duration of symptoms for two weeks. The third pattern of the disease course corresponds to critical patients who present with shock, sepsis, severe hypoxemia, multiple organ failure, need for mechanical ventilation, and intensive treatment. About 5% of cases will have this course and it reaches three weeks of duration. In these cases, there is initially a high viral load, followed by a decrease in viral load and immune system overactivation, at a second moment (Lescure et al., 2020).

The clinical manifestations of COVID-19 ranged from mild non-specific symptoms to severe pneumonia with organ function damage (Ge et al., 2020; Guan et al., 2020). In recent meta-analysis, the more common symptoms were fever, cough and dyspnea. Many case reports (in China, Europe and EUA) described the same clinical manifestations with some variations of frequency, as shown in **Table 1** (Adlhoch et al., 2020; Ge et al., 2020; Guan et al., 2020; Hirsch et al., 2020; Rodriguez-Morales et al., 2020; Spinato et al., 2020.

**Table 1:** Clinical manifestations of COVID-19 according to several studies (Ge et al., 2020; Guan et al., 2020; Hirsch et al., 2020; Rodriguez-Morales et al., 2020; Coronavirus disease 2019 (COVID-19) pandemic: increased transmission in the EU/EEA and the UK—seventh update, 2020).

Manifestation	Frequency (%)
**Fever **	77.4–98.6
**Cough**	59.4–81.8
**Fatigue**	38.1–69.6
**Dyspneia**	3.2–55.0
**Myalgia**	11.1–34.8
**Loss of taste**	47.3–61.5
**Loss of smell**	34.0–80.0
**Sputum production**	59.4–81.8
**Headache**	6.5–33.9
**Sore throat**	28.0–56.0%
**Rhinorrhea**	29.5–43.1%
**Chest pain**	11.5–22.2%
**Hemoptysis**	1.2–3.5%
**Conjunctival congestion**	5.0–9.5%
**Diarrhea**	3.8–10.0%
**Nausea and vomiting**	5.0–39.6
**Abdominal pain**	8.2–17.7
**Proteinuria**	34.0–65.0
**Acute renal injury**	3.0–36.6
**Tromboembolic event**	9.0–50.0
**Cardiac diseases**	7.0–44

The COVID-19 affects multiple organs and presents several non-specific laboratorial findings, as abnormal levels of active inflammatory markers (C-reactive protein, interleukin 6, erythrocyte sedimentation rate, lactic dehydrogenase), coagulation related dysfunction (thrombocytopenia and increased d-dimer), and lymphopenia (Rodriguez-Morales et al., 2020).

As previously mentioned, respiratory tract symptoms represent the most common clinical presentation of COVID-19. However, renal damage has shown to be also frequent and of prognostic relevance. Such involvement is variable, including, for example, asymptomatic hematuria and proteinuria, collapsing glomerulopathy, acute diffuse glomerulonephritis, acute kidney injury (e.g. acute tubulointerstitial nephritis), endothelial injury and renal microvessel thrombosis (Fanelli et al., 2020; Farkash et al., 2020; Larsen et al., 2020; Naicker et al., 2020). A study from China showed that proteinuria was present in 43.9% of the inpatients and hematuria in 23.7% (Cheng et al., 2020). Acute kidney injury was observed in 3.0 to 30% of the patients with COVID-19 (Diao et al., 2020; Durvasula et al., 2020; Farkash et al., 2020; Naicker et al., 2020). Data from New York showed that 36,6% of the inpatients developed acute kidney injury (AKI) and 14.3% required renal replacement therapy (Hirsch et al., 2020).

There is already evidence that AKI in COVID-19 can be caused by direct renal infection, but frequency and clinical significance of this disease mechanism is still not established (Farkash et al., 2020).

## SARS-CoV-2

Coronavirus is a large family of viruses. There are hundreds of coronaviruses, most of them hosted by animals, including pigs, camels, bats and cats. Sometimes these viruses can adapt, migrating from one host species to another, thus causing diseases in humans.

In humans they usually cause mild to moderate diseases of the upper respiratory tract, such as the common cold. SARS-CoV-2 is the pathogen that causes COVID-19, identified as the seventh type of coronavirus to infect humans (Zhu et al., 2020). The other six types of coronavirus are known to cause human diseases, including SARS-CoV and MERS-CoV (Su et al., 2016), four of them (229E, OC43, NL63 and HKU1) cause mild manifestations. According to genomic characteristics, coronavirus is separated into four gender: α-CoV, β-CoV, γ-CoV, and δ-CoV (Su et al., 2016; Zhu et al., 2020). The sequencing of the new coronavirus isolated from a sample of the lower respiratory tract of a patient infected with COVID-19 showed that it belongs to the β-CoV19.

Coronaviruses look like a crown under electron microscopy. They are enveloped viruses with a single strand of positive RNA sense and among all viruses, they have the largest known RNA genome (Forni et al., 2017). All CoV share the same genomic organization and gene expression pattern, encoding proteins: Spike (S), Envelope (E), Membrane (M) and Nucleocapsid (N) (Forni et al., 2017). The S protein plays an essential role in binding to host receptors and is determinant to viral tropism and transmission capacity. The SARS-CoV-2 S protein has 10 to 20 times more affinity to bind to the angiotensin converting enzyme 2 (ACE2) compared to SARS-CoV (Wrapp et al., 2020). ACE2 is essential to the virus entry in the host cell. After entering the cell, viral RNA is released into the cytoplasm and begins to be replicated by the cellular machinery (Perlman and Netland, 2009). The newly glycoproteins are inserted into the membrane of the Golgi endoplasmic reticulum and the nucleocapsid is formed by the combination of the genomic RNA and the Nucleocapsid (N) protein. Then, the viral particles from the Golgi fuse with the plasma membrane of the cell and the new viruses are released (de Wit et al., 2016).

A recent report showed that Acute Respiratory Discomfort Syndrome (ARDS) is the leading cause of death from COVID-19 (Huang et al., 2020). It is characterized by pleural effusion, depriving the lungs of oxygen receiving. ARDS usually occurs 8 days after appearing the first symptoms of COVID-19 (Wang D. et al., 2020). In one study, 15% of 41 patients infected with COVID-19 died of ARDS (Huang et al., 2020). ARDS is the most common immunopathological event in SARS-CoV-2, SARS-CoV, and MERS-CoV infections (Xu et al., 2020). Possible explanation to fast lung deterioration is the cytokine storm, one of the main mechanisms for ARDS. Uncontrolled systemic inflammation resulting from the release of large amounts of pro-inflammatory cytokines (IFN-α, IFN-γ, IL-1β, IL-6, IL-12, IL-18, IL-33, TNF-α, TGFβ, etc) and chemokines (CCL2, CCL3, CCL5, CXCL8, CXCL9, CXCL10, and others) by the effector immune cells in SARS-CoV infection (Cameron et al., 2008; Williams and Chambers, 2014; Channappanavar and Perlman, 2017; Huang et al., 2020) will cause a violent attack of the immune system on the body, causing ARDS and multiple organ failure, damage to the lungs, kidney and heart, which can lead to death in severe cases of SARS-CoV-2 (Shimabukuro-Vornhagen et al., 2018; Xu et al., 2020). The development of an effective approach to modulate the immune response or minimize cytokines production is crucial to reduce both worsening and mortality rate of SARS-CoV-2. Also, identifying safe and effective treatment options are urgent requirements (Zhou et al., 2020).

## Cathepsin L

Cathepsins (“to digest” in Greek, katahepsein) (Zhou et al., 2015) belong to a family of proteases that are responsible for recycling cellular proteins inside of the lysosomes (Turk et al., 2000; Conus and Simon, 2008). These proteases are comprised of serine, aspartate and cysteine peptidases and exhibit endo- or exopeptidase activities (Cao et al., 2017). They are synthesized as inactive proenzymes, or zymogens, and are activated by removing the predomain and prodomain during their translocation from the endoplasmic reticulum to the Golgi apparatus and to the lysosomal and endosomal compartments (Liu et al., 2018). For their optimal activity, cathepsins require reduced pH (between 4.5 and 5.0), such as found in the lysosome (Fonović and Turk, 2014; Liu et al., 2018). Moreover, cysteine protease cathepsins have traditionally been considered as lysosome-restricted proteases that mediate proteolysis of unwanted proteins (Cao et al., 2017; Liu et al., 2018). The CatL, a cysteine protease, is a matrix-degrading enzyme known to be increased in chronic inflammation (Cao et al., 2017). It is expressed in all tissues and cell types and the primary function of cysteine cathepsins is proteolysis of protein antigens generated by pathogen endocytosis (Maehr et al., 2005; Liu et al., 2018).

In humans, cathepsins have a role in various physiological processes, like apoptosis, antigen processing, extracellular matrix remodeling and MHC class II immune responses. Elastolytic cysteine proteases are mobilized to the cell surface of macrophages and other cells under inflammatory conditions, which lead to accelerated collagen and elastin degradation, exacerbating inflammation and tissue damage (Cao et al., 2017; Liu et al., 2018).

Although CatL is commonly recognized as a lysosomal protease, it is also secreted (Roth et al., 2000). The excessive secretion of cysteine cathepsins is implicated in various pathological states and is often linked with inflammation (Fonović and Turk, 2014). Thus, large amounts of CatL could be activated in inflammatory cells under inflammatory conditions. Furthermore, this enzyme plays an important role in the development, progression and metastasis of cancer and therefore its inhibition could prove useful in the management of this disease (Mittal et al., 2011). The plasma CatL has been proposed as a marker for pancreatic cancer (Singh, 2014).

The correlation of CatL activity with markers of inflammation such as neutrophil count suggests that the release of activated CatL into circulation occurs in conjunction with ongoing local or systematic inflammation (Cao et al., 2017). In a study that developed a model of sepsis-induced acute renal injury, CatL was shown to play an important role in the initial inflammatory response by activating M1 macrophages (Hu et al., 2020), which are major contributors to inflammation and tissue damage during chronic inflammatory processes and which have the highest expression of cathepsin. Therefore, CatL could also participate in triggering the cytokine storm however data to support this hypothesis is still lacking. In addition, the activation of toll like receptor (TLR) increases the intracellular activity of cathepsins and stimulates their proteolytic capacity without changing mRNA expression [17]. Furthermore, CatL activates heparanase and contributes to the inflammatory cascade by macrophage activation (Garsen et al., 2016). Heparanase is an important factor that participates on viral infection processes (Koganti et al., 2020).

Because of their important roles in various diseases, Hopkins et al. (2018) showed evidence that the inhibition of CatL is detrimental to the infection, thus several cathepsins have been considered as valuable therapeutic target (Fonović and Turk, 2014; Hopkins et al., 2018).

It was demonstrated that CatL is important for SARS-CoV-2 to enter host cells. The infection is characterized by the binding of the virus to the receptor, which induces conformational changes in the viral S glycoprotein, followed by proteolysis by CatL in the endosomes.

### Cathepsin L and Spike Processing

**Figure 1:**
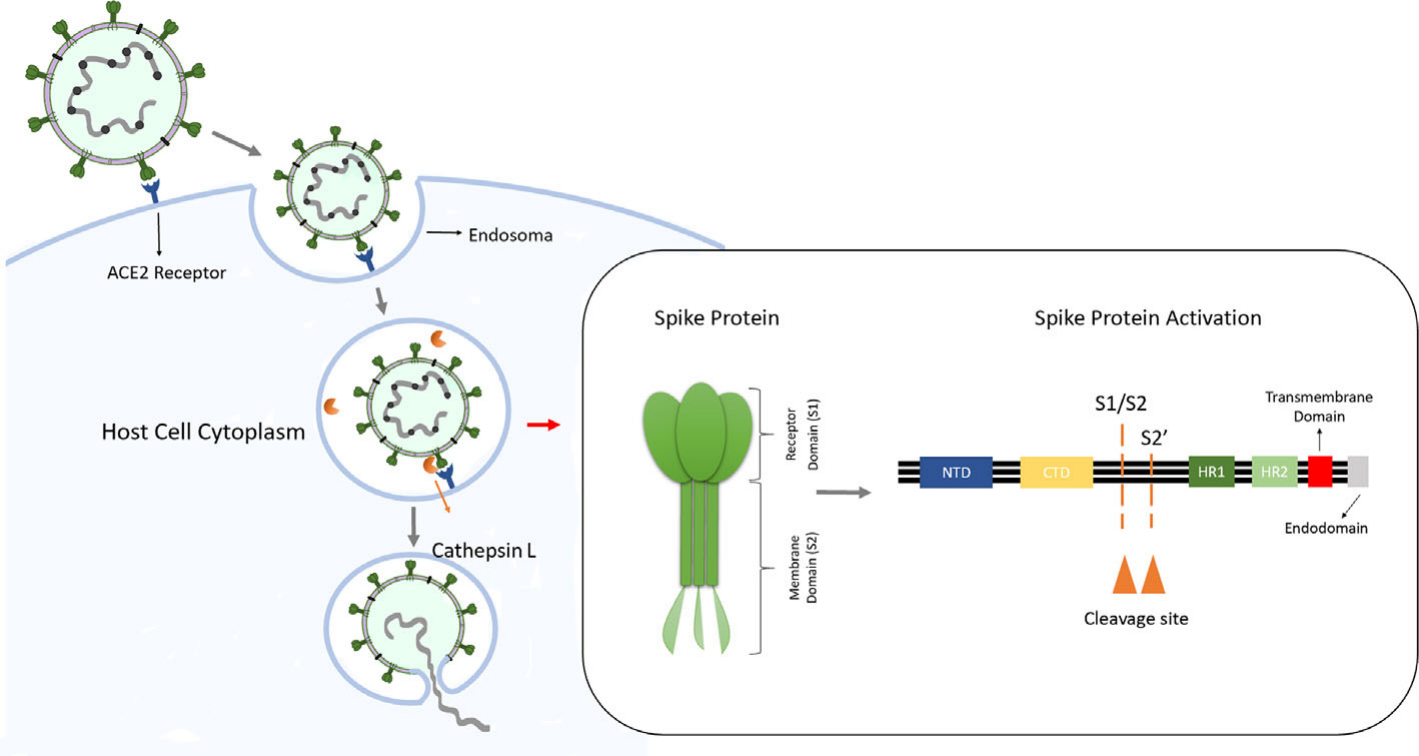
SARS-Cov infection into target cells, entry via endosomes. After interaction with ACE2 in the host cell, the virus can entry into the cell via endosomes. The spike protein is formed by subunits 1 and 2 and has two functions: first the S1 binds to ACE2 at the cell membrane followed by the S2 mediation of the fusion between Sars-Cov-2 and the endosome membrane, releasing the viral genome into the host cell. The fusion occurs after spike protein activation by host protease cathepsin L through the cleavage of S1/S2 border and the S2’s position.

In humans, the coronavirus infection occurs through the interaction between the viral S protein and ACE2 present in the host cell. The S protein is a trimeric glycoprotein formed by an ectodomain, a single-pass transmembrane anchor, and an intracellular tail. Each monomer of S protein is divided in two subunits: an attachment subunit (S1) composed of an N-terminal domain (NTD), which is involved in sugar bindings and a C-terminal domain (CTD) capable of recognizing the human ACE2. The subunit 2 (S2) contains the putative fusion peptide, the heptad repeats HR1 and HR2 and is involved in the viral membrane fusion. During the virus entry, S1 binds to ACE2 of the host cell and S2 fuses, allowing the genomes to enter the host cells (Kirchdoerfer et al., 2016; Wang N. et al., 2020) (**Figure 1**).

As a typical class I fusion protein, for the activation of the fusion potential of the S protein a cleavage by a host protease is required. The cleavage can be done from different stages of the virus infection cycle by host proteases as proprotein convertases (furin), extracellular proteases (elastase), cell surface proteases (TMPRSS2) and lysosomal proteases (CatL and CatB) (Li, 2016). The S protein activation occurs by two cleavage steps: first occurs a cleavage between the S1 and S2 domains, subsequently an additional cleavage at the S2 occurs promoting unmasking and activating of the fusion peptide (Smieszek et al., 2020).

Furin is a protein implicated in viral diseases, which cleaves some glycoproteins from the viral envelope and increases the viral fusion to host cell membranes (Moulard and Decroly, 2000; Izaguirre, 2019). The pathogenicity of some coronaviruses depends on the furin-like cleavage of the S protein (Coutard et al., 2020), which seems to worsen the symptoms of viral bronchitis, as well as increases viral pathogenicity (Cheng et al., 2019). In addition, the most pathogenic forms of the influenza virus present furin cleavage sites. A wide variety of cells express furin, which may explain their viral tropism (Kido et al., 2012). A previously described functional polymorphism, which affects the promoter region of the furin gene (c.-445C> T), increases the expression of this protein. Individuals carrying this variant apparently are at higher risk to develop chronic hepatitis B with poor prognosis (Lei et al., 2009).

Elastase is a serine protease secreted by the neutrophils when a viral infection happens (Barrett et al., 2004) and may induce pulmonary injury, which can contribute to acute respiratory distress syndrome, pulmonary fibrosis and chronic obstructive pulmonary disease (Kawabata et al., 2002; Hashimoto et al., 2008). The elastase these cells secrete is one of the proteolytic enzymes that can mediate the fusion activation the spike (S) protein of the coronavirus, modulating the fusion activation and the virus infection itself (Belouzard et al., 2010).

*TMPRSS2* gene encodes a transmembrane serine protease that regulates cell-cell and cell-matrix interactions. Several studies linked this protein to some types of cancer and viral infections (Choi et al., 2009; Stopsack et al., 2020). This gene is regulated by the androgenic hormones and it can be upregulated in prostatic cancer cells (Clinckemalie et al., 2013). The increased expression of *TMPRSS2* increases viral spike protein uptake (Heurich et al., 2014). Additionally, *TMPRSS2* can facilitate the SARS-CoV-2 to enter the host cell by two different mechanisms: 1) ACE2 activation, and 2) protein S activation (Clinckemalie et al., 2013; Heurich et al., 2014).

In the S protein of the SARS-CoV-2, the cleavage occurs between the residues Thr696 and Met697 in the S1-S2 domains, the same in the SARS-CoV, promoting the release of the virus’s genome into the host cell, indicating that CatL could act in this process (Smieszek et al., 2020). Recently Ou et al. (Ou et al., 2020) investigated, by in vitro experiments, the importance of CatL for the SARS-CoV-2 entry into the host cell and concluded that CatL inhibition decreases virus entry by more than 76%. Their results show that CatL is a pivotal strategy for the virus to enter the host cell via endosome. However, there was no complete blockage of the entry of SARS-CoV-2, indicating that protein S is also activated on the cell surface allowing the virus entry.

In the SARS-CoV infection, it has been shown that the infection mechanism can occur by endocytosis and the spike activation is processed by CatL (**Figure 1**) (Simmons et al., 2004; Simmons et al., 2005; Huang et al., 2006; Bosch et al., 2008; Ou et al., 2020). The study of Simmons and colleagues (Simmons et al., 2005) demonstrated that in the SARS-CoV infection, recombinant CatL was sufficient to activate S protein leading to membrane fusion, while the infection was blocked by specific inhibitors of this protease. These results are in agreement with the Huang et al. (2006), that showed SARS-CoV utilizes the CatL to infect ACE2 expressing cells.

### Cathepsin L as a Therapeutic Target

CatL is implicated in invasion and metastasis of tumors, inflammatory status, atherosclerosis, renal disease, diabetes, bone diseases, viral infection and other diseases. Functions of CatL depend on their subcellular localization: it is involved in cell death and inflammation in the cytoplasm, and it also regulates cell cycle in the nucleus and exert degradative roles in the extracellular environment (Pan et al., 2020).

The biological importance of CatL promoted several initiatives aiming to develop CatL inhibitors. In 1981, the first CatL inhibitor cystatin was isolated from Aspergillus and, today, a lot of molecules have been synthesized. The irreversible inhibitors include epoxy succinic acid, beta-lactams, vinyl sulfone and acyl hydrazine derivatives while the reversible inhibitors mainly include aliphatic, cycloketone, aldehyde and nitrile derivatives.

In previous studies with other caliciviruses, for example, CatL inhibitors and also chloroquine, significantly reduced the replication of some of those viruses. Confocal microscopy analysis showed that viral capsid proteins were retained in the endosomes in the presence of a CatL inhibitor or chloroquine during virus entry. Then it was suggested an important role of endosome maturation and CatL in the entry of caliciviruses, and CatL as a possible therapeutic target against calicivirus infection (Geleris et al., 2020).

Currently, there are no drugs available to treat SARS-CoV-2 infections. Once CatL has been demonstrated to be important to the virus entry and possibly exit during late stages of infection, drugs exhibiting capacity of CatL inhibition could offer potential COVID-19 therapy, even considering they were not primarily developed for this purpose and their true role as COVID-19 treatment needs controlled clinical trials. However, some considerations are important: cathepsin inhibitors exert toxicity in cells, and broad-spectrum inhibition of CatL may lead to some unpredictable side effects due to the complex function of cathepsins in normal physiological processes (Li et al., 2017).

### Amantadine Hydrochloride

High throughput drug screens are conducted in order to find a possible treatment. Using this approach, Smieszek and colleagues (Smieszek et al., 2020) demonstrated that the agent amantadine hydrochloride, used to treat influenza A, inhibits CatL mRNA expression, also possibly altering the CatL functional environment.

### Teicoplanin

Teicoplanin is an antibacterial drug previously used to treat virus infections caused by influenza virus, hepatitis C virus, HIV, Ebola, flavivirus, as well as MERS-CoV and SARS-CoV. Teicoplanin acts as a CatL inhibitor, blocking the S protein cleavage (Zhou et al., 2016; Yousefi et al., 2020).

### Chloroquine and Hydroxychloroquine

Chloroquine and hydroxychloroquine are aminoquinolines usually prescribed for the treatment of malaria and rheumatic diseases, as systemic lupus erythematosus and rheumatoid arthritis. One of the well-known actions of cloroquine is to increase endosomal pH, which can lead to inhibition of CatL activation, and consequently the activation of protein S in the endosomes would be impaired (Gonzalez-Suarez et al., 2011). During the COVID-19 pandemics, it was suggested that these drugs could be effective to treat such infection based on both antiinflammatory and antiviral effects. Nevertheless, a clinically relevant antiviral action against SAR-CoV-2 was not demonstrated until now. In fact most recent studies recommend not using chloroquine or hydroxychloroquine for treatment of COVID-19 regardless of the disease phase (Peters et al., 2020).

### Heparin

Mycroft-West et al. (2020) demonstrated that heparin and its derivates bind to the SARS-CoV-2 spike protein and inhibit viral infection. Additionally, it has been shown that glycosaminoglycans bind to spike S1/S2 proteolytic cleavage site (Kim et al., 2020b). Owing to the fact that CatL is important for viral invasion (Simmons et al., 2004; Simmons et al., 2005; Huang et al., 2006; Bosch et al., 2008; Ou et al., 2020) and that heparin was described as an inhibitor of the CatL activity (Higgins et al., 2010), the antiviral properties observed for heparin could be related to impaired S1/S2 proteolytic during SARS-CoV-2 cell invasion.

**Figure 2:**
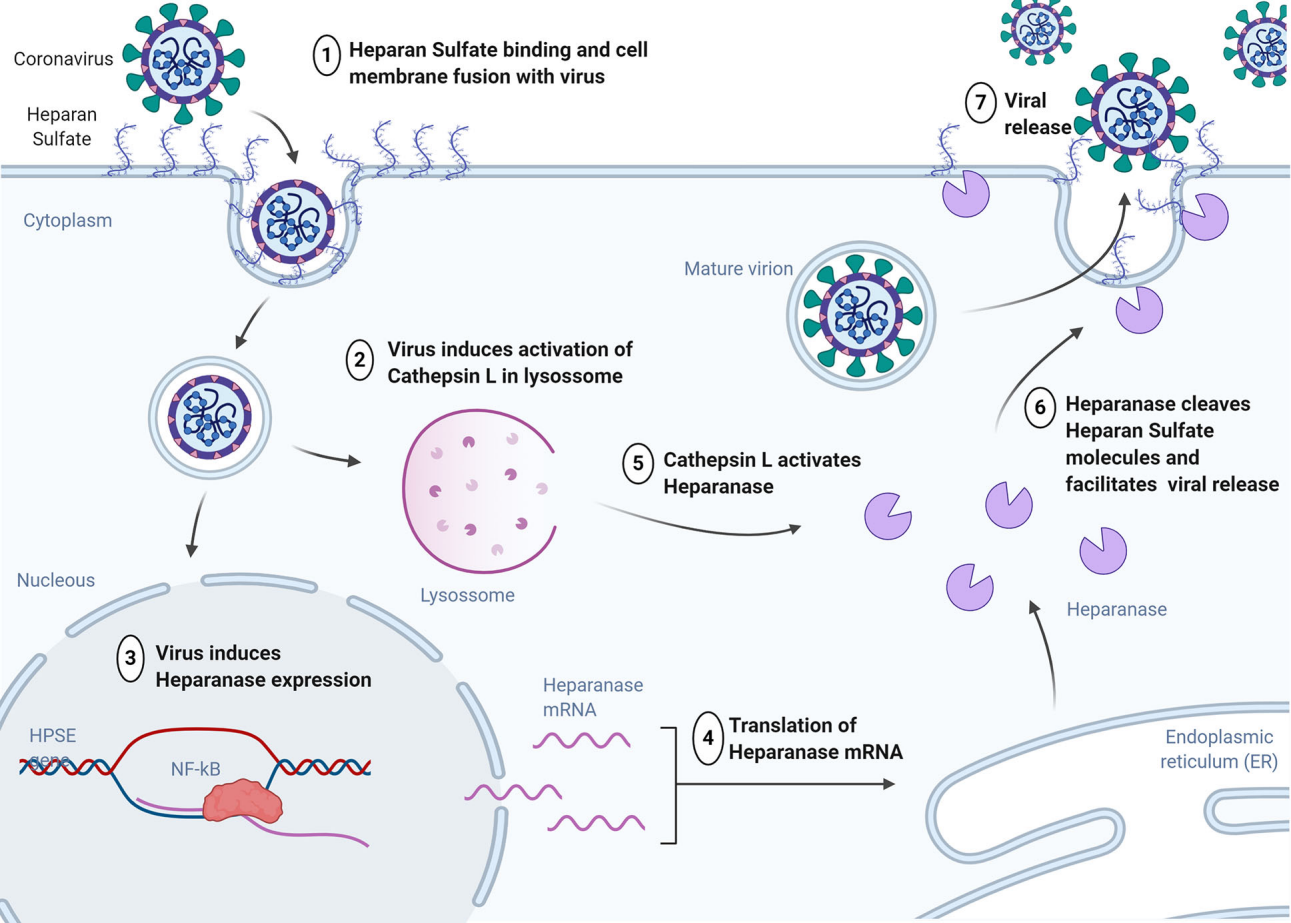
Heparan Sulfate expression increases during the initial stage of viral infection to enhance viral attachment to cells. Virus inside of cell induces activation of cathepsin L in lysosome and promotes upregulation of heparanase expression through the NF-kB signaling pathway. Heparanase protein is activated by Cathepsin L and is translocated to the cell surface for the removal of heparan sulfate to facilitate viral release. HPSE, heparanase gene; NF-kB, nuclear factor kappa beta. (Created in Biorender.com).

Since long time it is known that heparan sulfate could act as a major binding locus for a large number of human viruses, including dengue virus, hepatitis C virus, human immunodeficiency virus, human papilloma virus and essentially all herpes viruses (Chen et al., 1997; Feldman et al., 2000; Esko and Lindahl, 2001; Giroglou et al., 2001; Barth et al., 2003; Rivara et al., 2016). Recently, studies have shown that heparan sulfate and heparanase are major regulators of viral release (**Figure 2**) (Vlodavsky et al., 2020).

Despite there are no studies with heparanase and SARS-CoV infection, its inhibition could be an alternative pathway to block its entry and release from host cells. In addition to the inhibition of CatL activity (Higgins et al., 2010), the therapeutic potential of heparin could also be linked to the fact that it is a substrate for heparanase and therefore its binding to this enzyme could inhibit degradation of heparan sulfate (Nasser et al., 2006).

Kim et al. (2020a) using the technique of surface plasmon resonance, demonstrated the existence of a glycosaminoglycan (GAG)-binding motif at S1/S2 proteolytic cleavage site in the SARS-CoV-2, and hypothesized that GAGs from the host cells could interact with the coronavirus, facilitating the virus to enter. This study shows that virus (GAG)-binding motif binds more tightly to immobilized heparin, which supports the role of heparin among the COVID-19–infected patients.

### Other Therapeutic Options

It is of note that so far except for supportive and intensive care, no standard medical therapy has been validated.

In addition to the above cited, some old medications are under investigation to establish a treatment for COVID-19, including ivermectin (known to have antiviral actions), vitamin D (immunomodulatory and anti-inflammatory actions (Jakovac, 2020), tocilizumab (being tested as supplementary treatment for COVID-19 patients with cytokine release syndrome) (Jean and Hsueh, 2020), and remdesivir (that was superior to placebo in shortening the time to recovery in adults hospitalized with COVID-19 and lower respiratory tract infection (Beigel et al., 2020), among others.

## Genetics of CatL and Susceptibility to COVID-19

An important issue raised by the recent pandemic of SARS-CoV-2 is the identification of specific human gene variants contributing to enhanced susceptibility or resistance to viral diseases. Clinical variation in COVID-19 severity and symptomatic presentation highlighted the possible differences presented by host genetics.

CatL is a ubiquitously expressed endopeptidase whose gene (CTSL) is located at the 9q21-q22 position of chromosome 9 and encodes for a total of 333 amino acid peptide sequence (MW = 37,564 kDa) (Dana and Pathak, 2020). Human CatL is encoded by at least five mRNA variants with similar stabilities but different translation efficiencies, transcribed from the same gene. The CTSL is activated by a variety of growth factors, tumor promoters and second messengers (Turk et al., 2000; Honey and Rudensky, 2003; Schurigt et al., 2012; Li et al., 2017; Abbas et al., 2018). Cathepsin expression is regulated by natural cathepsin inhibitors, including papain-type cysteine proteases (Sui et al., 2016) and cystatins (Belouzard et al., 2012). Multiple mechanisms increase cysteine cathepsin expression in tumors, including alternative splicing of CatL transcripts (Mohamed and Sloane, 2006). Therefore, different virus, including SARS-CoV-2, could use similar mechanisms to overexpress cathepsins. In addition, as the SARS-Cov-2 virus uses CatL to infect host cells, certain genetic variants in the gene could in theory affect the propagation capacity of the virus, protecting or even making individuals or a population more susceptible to viral infection.

Although this is a viable hypothesis, little is known about the molecular variants of the CTSL and their physiopathological implications. In the Human Gene Mutation Database (HGMD), there is only one study deposited that evaluated a polymorphism in the CatL gene. In this study, Mbewe-Campbell et al. evaluated a functional polymorphism in the promoter region of the CatL gene, and concluded that patients carrying this polymorphism are prone to develop hypertension and cardiovascular problems (Mbewe-Campbell et al., 2012). Interestingly, Chapman et al. (1997) have shown that abnormal glycosylation caused by N-terminal point mutations is correlated with failure to sort mouse procathepsin L to lysosomes. Similar effect could be associated with the human CTSL gene, leading to variable cathepsin activity in different individuals. Therefore, more effort on population genetic sequencing, mainly on susceptible individuals, should be made to better understand the impact of the genetic variants in the human cathepsin gene in the susceptibility to SARS-CoV-2 and other viruses.

## Discussion

Cysteine cathepsins belong to a diverse family of proteases with individual members possessing distinct roles in a plethora of biological actions. CatL, one member of the family, as described here has an important role in the mechanisms of disease development in COVID-19, as SARS-CoV-2 utilizes CatL to infect host cells. Consequently, CatL inhibition represents a possible therapeutic target for COVID-19 treatment. However, an important issue raised by the recent pandemic of SARS-CoV-2 is the identification of specific human gene variants contributing to enhanced susceptibility or resistance to viral diseases. Clinical variation in COVID-19 severity and symptomatic presentation of some patients highlight the interindividual genetic differences, leading to the hypothesis that certain variants could protect or make individuals more susceptible to such viral infection, and related genetic profiles are under investigation. Despite their pivotal relevance to medicine and biology, only few large-scale genetic studies relating viral infections and human genetics are available (Kachuri et al., 2020). Although currently there are no drugs available to treat SARS-CoV-2 infections, one concern related to cathepsin inhibitors is their toxicity in cells. In addition, their broad-spectrum may lead to some unpredictable side effects due to the complex function of cathepsins in normal physiological processes (Li et al., 2017). The whole picture regarding how cysteine cathepsins function physiologically and specifically in the infection and progression of COVID-19 still requires much effort. The identification of the tissue and organs expressing CatL during the infection; the determination of the substrates cleaved by this cysteine protease and the interactions between CatL and other proteases and SARS-CoV-2 are still open and intriguing questions. In addition, the understanding of the whole picture concerning the genetic architecture of response to viruses may therefore provide new insight onto etiologic mechanisms of diverse viral diseases and CatL.

## Author Contributions

MTPR, PV, DEF, MGP, and FMC performed the literature search and wrote the manuscript under the supervision of JBP and GM-K. All authors contributed to the article and approved the submitted version.

## Funding

This work was supported by grants from the São Paulo State Foundation (Process 2014/27198-8 from João Pesquero and Process 2019/05266-5 from Gianna Mastroianni Kirsztajn).

## Conflict of Interest

The authors declare that the research was conducted in the absence of any commercial or financial relationships that could be construed as a potential conflict of interest.
